# Gait Characteristics Associated with Fear of Falling in Hospitalized People with Parkinson’s Disease

**DOI:** 10.3390/s23031111

**Published:** 2023-01-18

**Authors:** Manuela Uhlig, Tino Prell

**Affiliations:** 1 Department of Neurology, Jena University Hospital, 07743 Jena, Germany; 2 Department of Geriatrics, Halle University Hospital, 06120 Halle, Germany

**Keywords:** fear of falling, Parkinson’s disease, geriatric patient, gait analysis, stride length, wearables

## Abstract

Background: Fear of falling (FOF) is common in Parkinson’s disease (PD) and associated with distinct gait changes. Here, we aimed to answer, how quantitative gait assessment can improve our understanding of FOF-related gait in hospitalized geriatric patients with PD. Methods: In this cross-sectional study of 79 patients with advanced PD, FOF was assessed with the Falls Efficacy Scale International (FES-I), and spatiotemporal gait parameters were recorded with a mobile gait analysis system with inertial measurement units at each foot while normal walking. In addition, demographic parameters, disease-specific motor (MDS-revised version of the Unified Parkinson’s Disease Rating Scale, Hoehn & Yahr), and non-motor (Non-motor Symptoms Questionnaire, Montreal Cognitive Assessment) scores were assessed. Results: According to the FES-I, 22.5% reported low, 28.7% moderate, and 47.5% high concerns about falling. Most concerns were reported when walking on a slippery surface, on an uneven surface, or up or down a slope. In the final regression model, previous falls, more depressive symptoms, use of walking aids, presence of freezing of gait, and lower walking speed explained 42% of the FES-I variance. Conclusion: Our study suggests that FOF is closely related to gait changes in hospitalized PD patients. Therefore, FOF needs special attention in the rehabilitation of these patients, and targeting distinct gait parameters under varying walking conditions might be a promising part of a multimodal treatment program in PD patients with FOF. The effect of these targeted interventions should be investigated in future trials.

## 1. Introduction

Falls, and fear of falling (FOF), are common and serious problems in people with Parkinson’s disease (PD) [1,2]. FOF has been defined as ongoing concerns about falling, low fall-related self-efficacy, fearful anticipation of falling, and activity avoidance [3,4]. FOF restricts mobility, social participation, and quality of life [5,6]. FOF predicts future falls and therefore is relevant to consider FOF for fall risk assessment [7,8,9,10,11]. 

PD gait is often characterized by reduced step length, reduced gait speed, delayed gait initiation, shuffling, and freezing of gait (FOG) [12]. Gait changes can be assessed by clinical observation, standardized assessments, and with objective quantitative gait analysis. Given the complexity of gait, subtle changes can be difficult to capture with clinical observation. Therefore, sensor-based technologies often including accelerometers and gyroscopes are promising tools to quantify gait patterns [13]. Objective gait analyses can improve our understanding of gait. Another advantage of wearable sensor-based gait analysis is that it does not require a gait laboratory. In the last years, several studies demonstrated how objective gait analyses aid in the diagnosis, symptom monitoring, therapy management, rehabilitation, fall risk assessment, and prevention in PD [14,15,16]. For example, gait parameters including gait speed may be altered years before PD diagnosis [17]. Findings from gait analysis can help to distinguish PD subtypes, predict the risk of falling and increase the sensitivity of classical clinical fall risk factors to discriminate fallers from non-fallers in PD [18,19,20]. In addition, wearables can also detect changes in PD symptoms due to treatment adaptation and rehabilitation [21,22].

Of note, PD gait patterns can be influenced by non-motor symptoms [23,24]. This is especially true for FOF. FOF in PD was found to be associated with impaired postural control, one-leg stance time, timed-up-and-go, Berg balance scale, 6-min walking, and the motor score of the Unified PD Rating Scale (UPDRS) [8,10,25,26,27]. However, these findings are restricted to clinical or semi-quantitative ratings. In contrast, objective quantitative gait analysis can provide additional and reliable insights into gait characteristics that are related to PD or PD symptoms [28,29] and might therefore improve our understanding of FOF-related gait changes in PD. For example, Bryant et al. analyzed 79 patients with PD from a specialized outpatient clinic and found that gait speed and stride lengths were poorer in people with a high level of FOF [8]. Moreover, FOF influences turn-to-sit transition [7] and turning metrics in PD [30]. In *de novo* PD, FOF influences backward gait speed, but not the forward gait or dual-task gait speed [31]. However, studies using objective gait analyses were only performed in younger, community-dwelling PD patients or outpatients [8,30,31]. Less is known about older and acutely hospitalized PD patients. It is important to close this gap, as the proportion of hospitalized PD patients is growing in Germany [32,33,34]. 

Therefore, this study aims to investigate the relationship between FOF and PD gait characteristics in acutely hospitalized neurogeriatric PD patients, in order to gain a deeper understanding of FOF-related gait in this vulnerable patient cohort. In particular, we aim to 1) describe patterns of FOF in people with PD admitted to the hospital for specialized treatment, and 2) to study the association between distinct gait parameters, clinical parameters, and FOF in this cohort. This can help to propose gait parameters that may be studied further in interventional trials, because gait difficulties may be promising targets for the effective treatment of FOF in advanced PD [26]. These findings could then be used in further studies, for example, to treat and monitor anxiety-associated gait disorders in PD patients.

## 2. Materials and Methods

### 2.1. Subjects and Clinical Assessment

This cross-sectional study recruited 79 participants with PD from the ward of the Department of Neurology, Jena University Hospital, Jena, Germany. All patients gave written informed consent. The study was approved by the local Ethics Committee and has been performed in compliance with the Declaration of Helsinki.

Inclusion criteria: PD diagnosis according to Movement Disorder Society’s (MDS) diagnosis criteria, admission to hospital for PD multimodal complex treatment [33], and the ability to walk 50 m without personal assistance.

Exclusion criteria: non-PD-related gait impairment, spasticity, cerebrovascular disorders, neuropathy, deep brain stimulation, levodopa/carbidopa enteral infusion, apomorphine infusion.

In Germany, many people with PD are treated in a multidisciplinary PD inpatient treatment concept called PD multimodal complex treatment [33,34]. In addition to pharmacological adjustments, multimodal complex treatment includes inter-professional treatment by physiotherapists, occupational therapists, speech and language therapists, and psychologists. PD multimodal complex treatment is an integrated part of the German health insurance system and takes place in accordance with the requirements of the Operation and Procedure Classification System as an official coding system for medical procedures. 

### 2.2. Assessments

All assessments were conducted during the medication ON phase and at the beginning of multimodal complex treatment (first or second day after admission to the hospital). The following explanatory parameters were collected:

Age (metric, years), sex (nominal, male/female). PD-related parameters: disease-duration (metric, years); motor and non-motor symptoms: MDS-sponsored revision of the UPDRS III (MDS-UPDRS III, metric) [35], the revised non-motor symptoms questionnaire (NMS-Quest, metric) [36], Hoehn & Yahr stage (multi-nominal, stage I to V), timed-up-and-go test (metric, sec) [37], history of falls within the previous 6 months (nominal, yes or no), freezing of gait (nominal, present or absent), and use of walking aid (nominal, yes or no). In addition, cognition (Montreal cognitive assessment; MoCa, metric) [38] and depressive symptoms (Beck’s depression inventory; BDI II, metric) were assessed.

FOF was assessed using the Falls Efficacy Scale International (FES-I, metric) (α = 0.94) [39]. The FES-I is a self-report questionnaire with a four-point scale, where the respondents answer how concerned they are about the possibility of falling in relation to 16 different activities (1 = not at all concerned to 4 = very concerned). The total FES-I ranges from 16 to 64, with higher values indicating more concerns about falling. FES-I total scores were categorized into three groups: low (16–19 points), moderate (20–27), and high concerns about falling (28–64), according to previous works [40,41].

### 2.3. Gait Assessment and Test Protocol

Participants were instructed to walk at their preferred speed on a straight and flat 50 m-long hallway at the ward of the Department of Neurology and were asked to turn at the respective end of the hallway without stopping. All participants were guarded by the author M.U. to prevent falls (M.U. walked behind the patient). Spatiotemporal gait parameters were automatically recorded by a validated mobile gait analysis system (RehaGait^®^, HASOMED GmbH, Magdeburg, Germany) [42,43]. RehaGait^®^ consists of two inertial sensors attached to the shoes and streams raw data to a smart device application for real-time gait parameter calculation. A rule- and threshold-based pattern recognition algorithm was used to detect gait events (heel strike, full contact, heel off, toe off, etc.) [44] and a zero velocity assumption at full contact was used to minimize sensors integration drifts [45]. For the analysis, the initial stride, and all turning strides, including the stride before and after every turn, were excluded. The first 25 strides not excluded by the algorithm were used for this analysis. The following spatiotemporal gait parameters were recorded:

- Stride duration (s)
- Stride length (m)
- Speed (m/s)
- Cadence (steps/min)
- Toe clearance (m)
- Variability spatial (%)
- Variability temporal (%)

### 2.4. Statistical Analysis

The SPSS statistical computer package (version 25.0; IBM Corporation, Armonk, NY, USA) and JASP (version 0.16) were used for all statistical analyses. Prior to statistical analysis, data were checked for outliers and normality using the Shapiro-Wilk’s Test (*p* < 0.05). Descriptive analyses were used to describe clinical and gait characteristics. Correlations between FES-I, clinical variables, and gait variables were tested using Spearman correlation. To determine factors associated with FES-I we used stepwise multiple linear regression (Akaike information criterion as selection criterion). The explanatory variables entered in the model were the variables that significantly correlated with the FES-I in the univariate analyses. Multicollinearity was observed for several gait parameters as indicated by a variance inflation factor above 10; correlations are given in Table 1. Thus, only temporal variability, spatial variability, toe clearance, cadence, and speed were used as gait parameters in the regression analyses. 

## 3. Results

### Descriptives

Detailed clinical characteristics and gait parameters of participants are given in Table 2.

During testing, 63 participants walked without any assistive device, 13 walked with a wheeled walker (in German called “Rollator”), and 3 walked with a cane. Overall, 40 (50.6%) of the participants reported at least one fall in the last 6 months. According to the FES-I, 18 persons (22.5%) reported low concerns, 23 (28.7%) reported moderate concerns, and 38 (47.5%) reported high concerns about falling. 

**Figure 1:**
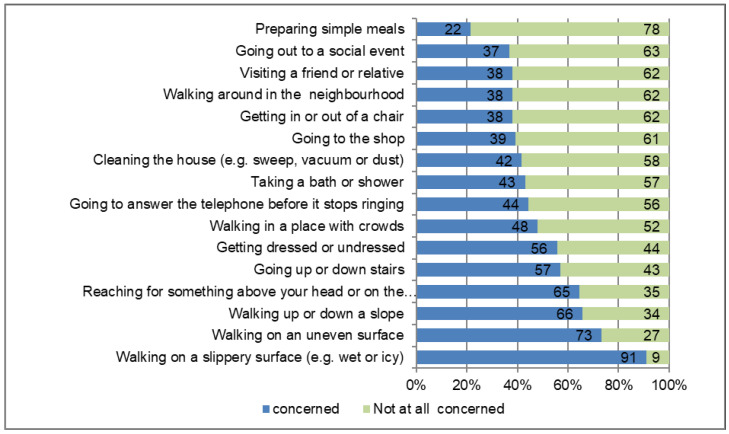
Item level of the Falls Efficacy Scale International (FES-I).

On the FES-I item level, most people reported FOF when walking on a slippery surface (e.g., wet or icy), on an uneven surface, or up or down a slope (Figure 1).

In the univariate analyses, the FES-I correlated with different clinical variables and gait parameters (Table 3). Higher FOF was associated with female sex, higher Hoehn & Yahr stage, poorer motor function (higher MDS-UPDRS III), presence of FOG, depressive symptoms (higher BDI), use of a walking aid, and falls in the past 6 months. 

Among the gait parameters, stride length, speed, toe clearance, and temporal gait cycle variability correlated with the FES-I (Table 3). 

We then calculated two regression models. In the first model, when only the gait parameters were entered as independent variables (i.e., temporal variability, spatial variability, toe clearance, cadence, speed), after stepwise regression only speed remained in the final model and explained 18% of FES-I variance (corrected R^2^ = 0.18, *F*(1, 77) = 18.3, *p* < 0.001). In the second model, we adjusted for clinical and demographic covariates and entered the independent variables that significantly correlated with the FES-I in the univariate analyses (see Table 3). Here, previous falls, BDI, use of walking aids, speed, and FOG explained 42% of the FES-I variance (Table 4). 

A post hoc power analysis revealed that with a coefficient of determination of ^R^ = 0.42, a statistical power of 0.9, and a significance level of α = 0.05, one would need a sample size of n = 39 for a significant overall model with 10 predictors. Therefore, our sample size was sufficient for the performed analyses.

## 4. Discussion

In this study, we investigated which gait parameters derived from objective quantitative gait analysis, are associated with FOF in hospitalized geriatric PD patients. In summary, FOF was related to previous falls, depressive symptoms, and the use of walking aids; we found that among the gait parameters, only speed was found to be associated with FOG. 

This is in line with a former study where both lower gait speed and stride length were associated with FOF in PD patients with a mean age of 69 years and a disease duration of 8.7 years [8]. However, due to multicollinearity, only speed (and not stride duration, stride length, or cadence) was entered into our model. In addition to speed, previous falls, depressive symptoms, walking aids, and FOG were found to be associated with FOF in our study. 

How can poorer gait performance in PD be related to FOF? It seems possible that these patients change their walking behavior after falls and due to FOF. Fall events within the last six months were the strongest independent variable for FOF in our study. This is consistent with studies showing that fall events in PD increase FOF [46,47]. FOF can lead to avoidance behaviors and restricts mobility [48,49] by a decrease in confidence in performing daily activities [46,50]. Every third fall increases the fear of walking in PD [51]. This fear, combined with less confidence in one’s abilities in everyday activities, could lead to or aggravate cautious walking. Gait speed in patients who had fallen was slower than in non-fallers [52]. Reduced gait speed in PD after falls as part of more cautious gait and FOF are significantly associated with previous falls [2,10,47,52]. A more cautious gait, which can also be observed in healthy older adults in general [53], is characterized by reduced gait speed, reduced step length, and lower toe clearance in order to be as “close as possible” in contact with the floor. This may be of greater concern due to the common postural instability in PD [54]. These interdependent factors may partially explain our results of walking more cautiously at slower speeds, reduced stride length, and reduced toe clearance.

In addition, our study also showed that several clinical parameters are associated with FOF. The association between FOG and previous falls is in line with earlier studies [8,55]. Furthermore, the associations between FOF, FOG, and depression are in agreement with earlier studies in other PD cohorts. In a previous study of 130 participants with PD [56], it was shown that those who experienced FOG while walking reported more falls in the past compared to PD patients without FOG. The same study also reported the occurrence of more intense depressive symptoms in PD with FOG compared to PD without FOG. A study by Franzén et al. [57] supports our findings on the influence of depressive symptoms on FOF. Finally, our study demonstrated the connection between the use of a walking aid and FOF in PD. The subjects in our study mostly used a cane or a wheeled walker. An association between FOF and walking aids is also known in older people not having PD [58]. 

Since FOF has been reported to be a significant predictor of future falls and reduced quality of life in PD [5,7,46,48,49], a better understanding of gait parameters associated with FOF may help to design effective treatment strategies for this vulnerable cohort [8,9,10,11]. However, reflecting critically on the results of our study, quantitative gait analysis has, in our opinion, little added value for understanding FOF in the cohort studied. Thus, no specific abnormalities were shown in relation to FOF, except for reduced gait speed (and consecutively reduced stride length), so that no new specific therapeutic options can be derived from this. Certainly, it seems reasonable for this vulnerable cohort of hospitalized PD patients to use a multimodal therapy regimen that targets both walking speed increase and clinical parameters (depression, FOG). The magnitude of the effect of an intervention that increases gait speed on FOF needs to be tested in future randomized trials. Regarding the therapy of people at risk of falling with PD and FOF, the results of the FESI item analysis are also interesting. Here, most people reported fear when walking on a slippery surface (e.g., wet or icy), on an uneven surface or up or down a slope. This can be a basis for tailored interventions with a special focus on these walking conditions (e.g., by forced training on uneven surface instead of walking on ground floor) within a multiprofessional and multimodal treatment.

Our study has limitations. First, we focused on straight walking on a flat corridor [30]. It may be promising to evaluate gait in more complex settings and movement behaviors such as turning and transfers or at home. Second, this study focused on gait parameters that are relevant for current rehabilitation approaches for PD [59,60]. There are more potentially independent gait parameters extractable with such an inertial measurement unit-based technique [61] and it is possible that a more refined analysis approach could unveil additional associations between specific gait impairments and FOF. We do acknowledge that there may still be other influential factors for FOF that deserve consideration, such as the level of physical activity and physical environmental barriers. Another limitation is that we cannot make causal statements with a cross-sectional dataset. 

In conclusion, the successful use of wearable devices for assessing mobility can be advantageous for both practitioners and scientists [62]. Gait characteristics obtained by wearables can be used to support tailored intervention rehabilitation and therapy plans [19,21,29,63]. However, one has to keep in mind that distinct motor- and non-motor features have to be considered when investigating gait and factors associated with gait disturbances in PD. With this study, we provided data about FOF and gait for an underrepresented cohort of acutely hospitalized PD patients. Our study suggests that FOF is closely related to gait changes in hospitalized PD patients, and thus, FOF needs special attention in the rehabilitation of these patients.

## Figures and Tables

**Table 1:** Correlation Matrix: Spearman correlations between gait parameters.

	1	2	3	4	5	6
1 Stride duration (s)	—					
2 Stride length (m)	−0.3677 ***	—				
3 Speed (m/s)	−0.6329 ***	0.9413 ***	—			
4 Cadence (steps/min)	−0.9993 ***	0.3666 ***	0.6315 ***	—		
5 Toe clearance (m)	−0.1284	0.7531 ***	0.6535 ***	0.1310	—	
6 Variability spatial (%)	0.0369	−0.6053 ***	−0.4811 ***	−0.0356	−0.5238 ***	—
7 Variability temporal (%)	−0.2139	0.2605 *	0.2949 **	0.2086	0.1039	0.1227

* *p* < 0.05, ** *p* < 0.01, *** *p* < 0.001.

**Table 2:** Demographical and clinical characteristics of the entire cohort (N = 79).

	Median	Mean	SD	IQR
Age (years)	74	72.76	7.33	8
Disease duration (years)	9	9.46	6.45	7
MDS-UPDRS III (0–132)	27	32.25	16.16	20
Montreal Cognitive Assessment (MoCA) (0–30)	22	21.86	4.20	7
Beck’s depression inventory II (BDI) (0–63)	11	12.20	8.63	10
Timed-up-go-test (s)	12.60	15.52	10.46	10.78
Falls Efficacy Scale International (FES-I) (16–64)	26	30.10	11.87	19
Stride duration (s)	1.120	1.168	0.135	0.165
Stride length (m)	0.930	0.953	0.241	0.335
Speed (m/s)	0.790	0.835	0.256	0.355
Cadence (steps/min)	106.780	103.924	11.084	14.930
Toe clearance (m)	0.115	0.111	0.027	0.040
Variability spatial (%)	10.720	11.790	6.666	7.940
Variability temporal (%)	5.080	5.554	2.600	2.775
		n	%	
Sex	female	30	38.0	
	male	49	62.0	
Hoehn and Yahr stage	1 2 3 4	7 16 37 19	8.9 20.2 46.8 24.1	
Presence of freezing of gait (FOG)	no FOG FOG	52 27	65.8 34.2	
Use of a walking aid	noyes	63 16	79.7 20.3	
Fall(s) within the last 6 months	no yes	39 40	49.4 50.6	

**Table 3:** Spearman correlation with fear of falling (FES-I).

Clinical Characteristics	r	*p*
Age (years)	0.034	0.769
**Sex, male**	**−0.247**	**0.028**
Disease duration (years)	0.100	0.382
**Hoehn and Yahr Scale**	**0.445**	**<0.001**
**Freezing of gait present**	**0.425**	**<0.001**
**MDS-UPDRS III**	**0.365**	**0.001**
Montreal Cognitive Assessment (MoCA)	−0.184	0.104
**Beck’s depression inventory II (BDI)**	**0.482**	**<0.001**
**Use of a walking aid**	**0.317**	**0.004**
**Fall(s) within the last 6 months**	**0.476**	**<0.001**
**Gait parameters**		
Stride duration (s)	0.162	0.153
**Stride length (m)**	**−0.441**	**<0.001**
**Speed (m/s)**	**−0.437**	**<0.001**
Cadence (steps/min)	−0.160	0.160
**Toe clearance (m)**	**−0.336**	**0.002**
Variability spatial (%)	0.125	0.274
**Variability temporal (%)**	**−0.261**	**0.020**

Significant correlation in bold.

**Table 4:** Multiple linear regression.

Variable	Coefficient	SE	*p*	beta
No falls	−6.949	2.226	0.003	0.343
BDI	0.458	0.157	0.005	0.297
No walking aid	−5.624	2.831	0.051	0.139
Speed	−9.144	4.707	0.056	0.133
No freezing of gait (FOG)	−3.780	2.385	0.117	0.088

The entered variables correlated significantly with Falls Efficacy Scale International (FES-I) in the Spearman correlation: gender, Hoehn & Yahr stage, Freezing of gait (no/yes), MDS-UPDRS III, Beck’s Depression Inventory II (BDI), gait speed, toe clearance, temporal gait variability, walking aids (no/yes), falls in the last 6 months (no/yes). The stepwise procedure with Akaike information criterion. Overall model *p* < 0.001, corrected R^2^ = 0.42.

## Data Availability

The data used to support the findings of this study are available from the corresponding author upon request for scientific purposes only.
